# Susceptibility Testing of Colistin for *Acinetobacter baumannii*: How Far Are We from the Truth?

**DOI:** 10.3390/antibiotics10010048

**Published:** 2021-01-05

**Authors:** Federica Sacco, Paolo Visca, Federica Runci, Guido Antonelli, Giammarco Raponi

**Affiliations:** 1 Department of Molecular Medicine, University of Rome “La Sapienza”, 00161 Rome, Italy; federica.sacco@uniroma1.it; 2 Clinical Microbiology Laboratory, University Hospital Policlinico Umberto I of Rome, 00185 Rome, Italy; guido.antonelli@uniroma1.it; 3 Department of Science, University of Rome “Roma Tre”, 00154 Rome, Italy; paolo.visca@uniroma3.it (P.V.); federica.runci@uniroma3.it (F.R.); 4 Department of Molecular Medicine, Laboratory of Virology and Pasteur Institute-Cenci Bolognetti Foundation, 00161 Rome, Italy; 5 Department of Public Health Sciences and Infectious Diseases, 00185 Rome, Italy

**Keywords:** *Acinetobacter baumannii*, intensive care unit, multidrug-resistant, colistin, susceptibility testing, broth microdilution, OXA enzymes, international clone lineages, sequence group

## Abstract

*Acinetobacter baumannii* is involved in life-threatening nosocomial infections, mainly in the intensive care units (ICUs), and often colistin may represent the last therapeutic opportunity. The susceptibility to colistin of 51 epidemiologically typed *A. baumannii* strains isolated in 2017 from clinical samples of patients hospitalized in the ICU of a tertiary care academic hospital was investigated. All isolates were carbapenem-resistant due to the presence of the *bla*_OXA-23_ gene in sequence group 1 (international clonal lineage II) and sequence group 4 (related to international clonal lineage II) isolates, and to the *bla*_OXA-24/40_ gene in sequence group 2 (international clonal lineage I) isolates. Vitek^®^2, agar diffusion, and broth microdilution tests showed major discordancy (≥2 dilution factors) in the minimum inhibitory concentration (MIC) values for colistin in 24 out of 51 isolates, resulting in erroneous reporting of qualitative susceptibility data for eight isolates. In growth kinetics experiments in the presence of colistin, five isolates grew with drug concentrations above the susceptibility breakpoint when incubated for >12 h, and three isolates showed the presence of heteroresistant subpopulations. This study highlights that the high frequency of isolation of carbapenem-resistant *A. baumannii* strains in high-risk infectious wards requires an accurate application of methods for detecting susceptibility to antibiotics, in particular to colistin, so as to ensure a correct therapeutic approach.

## 1. Introduction

*Acinetobacter baumannii* and species of the *A. baumannii* complex are Gram-negative coccobacilli involved in the etiology of nosocomial infections and manifesting an important antibiotic resistance. *A. baumannii* is the cause of a wide range of clinical manifestations, including hospital-acquired pneumonia (HAP), followed by urinary tract infections (UTIs), bloodstream infections (BSIs), and surgical site infections (SSIs), which occur preferentially in patients admitted to intensive care units (ICUs), where they have five to ten times higher risk of acquiring hospital infections [1].

Several predisposing factors have been identified, including previous antibiotic therapy, which can alter normal bacterial flora and select resistant strains. Furthermore, cross-transmission among patients and the hospital environment are the most likely sources of infection. Colonization or infection sustained by *A. baumannii* has been invoked as an independent risk factor of mortality [2], though the debate around the direct attribution of mortality to infections from *A. baumannii* is still open.

A number of *A. baumannii* isolates are highly resistant to most of the clinically available antibiotics [3]. Carbapenems have been used for decades as the antibiotics of choice in the clinical management of the infections caused by this organism. Yet, resistance to carbapenems emerged, leaving very few therapeutic options [4]. Class D (OXA-type) carbapenemases are the main enzymes responsible for this resistance. The OXA carbapenemases found in *A. baumannii* complex species belong to four subgroups: OXA-23-like, OXA-24-like, OXA-51-like, and OXA-58. The OXA-51-like subgroup is intrinsic to *A. baumannii*, occurring in most or all of the strains [5,6]. Other OXA enzymes have been detected with variable frequency in the major epidemic lineages, including the international clones (ICL) I, II, and III [7].

Recently, colistin has become one of the major therapeutic options in the management of carbapenem-resistant *A. baumannii* infections. However, colistin resistance has rapidly emerged in *A. baumannii* after reintroduction of this drug into the clinical practice [4]. To complicate the picture, observations have recently been made about the congruity of the results of the susceptibility profiles and the values of the minimum inhibitory concentration (MIC) obtained by automated diagnostic devices and diffusion methods [8]. These observations were assumed on the basis of the discontinuity of the diffusion of colistin in agar, which often renders untrustworthy the reading of the inhibition halos and of all the interpretations related to the diffusion method (for example E-test^®^ and various semiautomatic systems). As confirmation of this problem, a caveat for the interpretation of the susceptibility tests obtained through these techniques has been issued by the European Committee on Antimicrobial Susceptibility Testing (EUCAST), with the indication to perform sensitivity tests using the microdilution method, considered as a reference method [9,10,11,12].

One mechanism of colistin resistance involves reduced colistin binding to the bacterial outer membrane due to the charge modification of the lipopolysaccharide (LPS) moiety [13]. A second mechanism is the complete loss of lipid A, which also prevents the interaction of the antibiotic with the bacterial outer membrane [14]. In *A. baumannii* infections, another problem that may cause therapeutic failure is the emergence of resistance to colistin in subpopulations from an otherwise susceptible (MIC ≤ 2 mg/L) population, a phenomenon defined as colistin heteroresistance, often associated with previous use of the drug [15,16,17].

In this work, a collection of well-characterized, multidrug-resistant *A. baumannii* isolates obtained from clinical specimens of infected or colonized patients was used to compare the performances of both commercial and in-house prepared colistin susceptibility tests. Substantial discrepancies between an automated testing method and both agar diffusion and in-house broth microdilution methods were observed. Our findings highlight the need for confirmative colistin susceptibility testing by the broth microdilution method, especially in the case of borderline MICs, which are increasingly frequent among multidrug-resistant *A. baumannii*, so as to ensure a consistent therapeutic approach.

## 2. Results

### 2.1. Characteristics of A. baumannii Isolates

In the observational period of February to July 2017, 51 consecutive isolates of *A. baumannii* were obtained from different clinical samples of 32 patients (Table 1; Table S1) hospitalized in the ICU of a large teaching hospital in Rome, Italy. The *A. baumannii* isolates were responsible for 15 bloodstream infections (29.41%), including two cases of central venous catheter (CVC)-related bacteremia, 19 cases of ventilator-associated pneumonia (VAP) (37.25%), and 5 infections of other body sites (9.81%). Twelve isolates (23.53%) were involved in colonization of various body sites of patients without clinical signs of infection (Table S2).

All 51 isolates were unambiguously identified as *A. baumannii* by the Vitek^®^2 system (bioMérieux, Marcy l’Etoile, France) and MALDI-TOF (Bruker Daltonics, Bremen, Germany) with full agreement between the two methods.

### 2.2. Antimicrobial Susceptibility

Susceptibility tests performed with the Vitek^®^2 system revealed that all of the *A. baumannii* isolates showed MIC values above the resistance threshold for ten out of twelve of the antibiotics tested (amoxicillin/clavulanic acid; piperacillin/tazobactam; gentamicin; cefotaxime; ciprofloxacin; trimethoprim/sulfamethoxazole; imipenem; meropenem; ertapenem; fosfomycin). Moreover, 44 (86.27%) of the isolates were resistant to tigecycline and 6 (11.76%) were also resistant to colistin, disclosing extensively drug resistant (XDR) and pandrug-resistant (PDR) profiles [18], respectively (Table S3).

### 2.3. Identification of International Clonal Lineages and Association with Carbapenemase Genes

Multiplex PCRs designed to selectively amplify *ompA*, *csuE*, and *bla*_OXA-51-like_ genes [19] showed that the *A. baumannii* isolates belonged to three different lineages. It emerged that 13.73% of the isolates (7/51) belonged to the sequence group (SG) 2, associated with ICL I, as it amplified gene fragments of length equal to 702 bp (*csuE*), 559 bp (*bla*_OXA-51_), and 355 bp (*ompA*). Thirty-one isolates (60.78%) were assigned to SG1, associated with ICL II, as they amplified genes of length equal to 580 bp (*csuE*), 162 bp (*bla*_OXA-51_), and 343 bp (*ompA*). Thirteen strains (25.49%) belonged to SG4, genetically related to ICL II, as they amplified gene fragments of 559 bp (*bla*_OXA-51_) and 355 bp (*ompA*).

**Figure 1:**
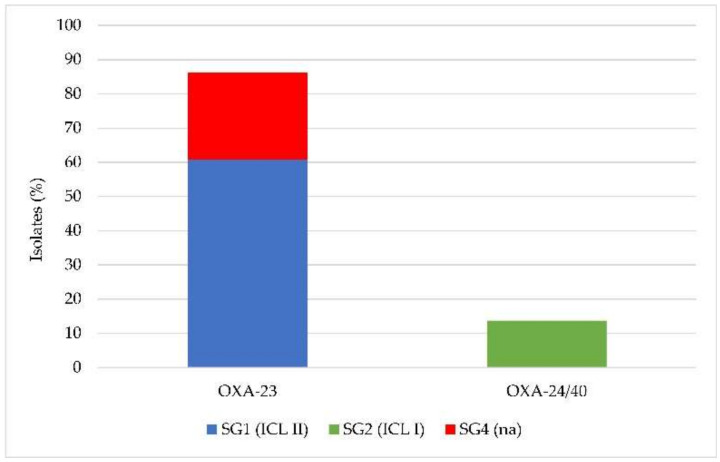
Presence of the *bla*_OXA-23_ or *bla*_OXA-24/40_ genes in the *Acinetobacter baumannii* isolates in relation to clonal lineage (na, international clones (ICL) not assigned).

Since all isolates were carbapenem-resistant, screening for the presence of class D carbapenemase genes was performed by multiplex PCR. Among the 51 *A. baumannii* isolates, 100% (51/51) carried the intrinsic *bla_OXA-51_* gene, confirming the taxonomic assignment as *A. baumannii*; 86.28% (44/51) tested positive for the *bla_OXA-23_* gene, and 13.72% (7/51) for the *bla_OXA-24/40_* gene, while none carried *bla_OXA-58_* or *bla_OXA-143_* genes. Therefore, the XDR and PDR phenotypes observed in these carbapenem-resistant isolates were invariably associated with the presence of oxacillinases, mainly the OXA-23 and OXA-24/40 enzymes. The association between SG1 (ICL II), SG2 (ICL I), SG4, and carbapenemase gene carriage is shown in Figure 1.

### 2.4. Comparative Analysis of Colistin Susceptibility Using Different Tests

Antibiotic susceptibility testing by Vitek^®^2 showed that 6/51 *A. baumannii* isolates (#24, 27, 34, 35, 48, and 51) were resistant to colistin (MIC ≥ 4 µg/mL). A parallel quantitative diffusion test using MIC Test Strips (Liofilchem srl, Roseto degli Abruzzi, Italy) showed that 12/51 isolates (#3, 6, 7, 9, 24, 25, 28, 34, 35, 46, 48, and 51) were resistant to colistin (MIC ≥ 4 µg/mL), and therefore a discordant interpretation between the two methods was found in 8/51 (15.68%) isolates (Table S4). The essential agreement (i.e., the % of isolates producing MICs that are within ± 1 doubling dilution) between Vitek^®^2 and the MIC Test Strips for colistin susceptibility testing was 54.90% (28/51 MIC results), far below the acceptability threshold (set at 90%).

To determine if there were any inaccuracies in reporting the colistin MIC values by Vitek^®^2, due to causes strictly referable to the instrument, colistin susceptibility was tested for all 51 *A. baumannii* isolates by the broth microdilution method, considered as the gold standard [8,9,10,11,12]. The broth microdilution test showed that 12/51 isolates (23.53%) were resistant to colistin. Furthermore, 11.76% (6/51) of the isolates showed discordancy between the values obtained with Vitek^®^2 and the microdilution method with discrepant MIC values of more than two dilution factors. By comparing colistin susceptibility results between the MIC Test Strips and broth microdilution, it emerged that 5.88% (3/51) of the strains showed a discrepancy between the two techniques with values greater than two dilution factors (Table S4). Relative to broth microdilution (golden standard), the essential agreement values for Vitek^®^2 and MIC Test Strips for colistin were 88.23% (45/51 MIC results) and 66.67% (34/51 MIC results), respectively (Table S4), thus below the acceptability threshold in both comparisons.

**Figure 2:**
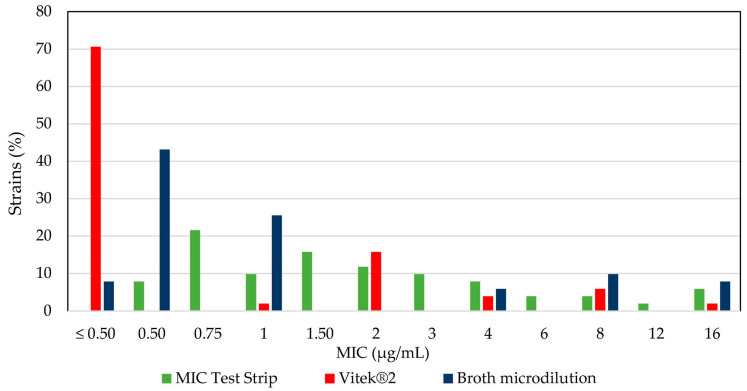
Percentage of *A. baumannii* strains susceptible to colistin at a given minimum inhibitory concentration (MIC), as determined by an MIC Test Strip, Vitek^®^2, and broth microdilution tests. The resistance breakpoint is ≥4 µg/mL.

A plot of the MIC values according to the test method provides evidence that colistin resistance is largely underestimated according to Vitek^®^2 results (Figure 2).

Of note, all isolates that presented discordant MIC values between Vitek^®^2 and the microdilution test belonged to ICL II (not shown).

### 2.5. Growth Kinetics of A. baumannii in the Presence of Colistin

**Figure 3:**
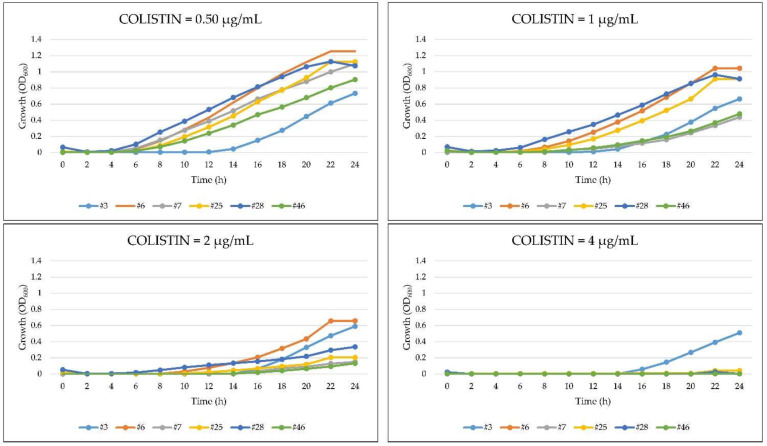
Growth profiles of six *A. baumannii* strains characterized by discordant MIC values for colistin (colistin-sensitive in the Vitek^®^2 assay but resistant in the broth microdilution test).

Spectrophotometric readings (OD_600_) provide a more objective assessment of bacterial growth compared with visual inspection of microplates. On this basis, six *A. baumannii* isolates (#3, 6, 7, 25, 28, 46) that resulted colistin-sensitive in the Vitek^®^2 assay but resistant in the broth microdilution test (Table S4) were selected to investigate their growth behavior in the presence of scalar concentrations of colistin (0.50–4 µg/mL). Colistin caused a dose-dependent reduction of the bacterial growth rate for all the tested strains, although the dose dependance and the extent of growth reduction differed between strains (Figure 3). However, all isolates showed appreciable growth in the presence of 2 µg/mL colistin, thus above the susceptibility threshold (≤2 μg/mL), and one isolate (#3) in the presence of 4 µg/mL colistin. These results are in agreement with those obtained in the broth microdilution test and provide evidence of its superiority with respect to the Vitek^®^2 assay, which categorized these isolates as colistin-sensitive (MIC ≤ 2 μg/mL).

### 2.6. Heteroresistance to Colistin in A. baumannii

The heteroresistance to colistin was evaluated in six *A. baumannii* isolates that displayed colistin susceptibility by Vitek^®^2 and resistance by broth microdilution (#3, 6, 7, 25, 28, 46; Table S4). Only three isolates, monitored for the growth at 24 and 48 h, formed colonies inside the inhibition halo of colistin MIC Test Strips. These colonies were tested for colistin susceptibility by all three methods and disclosed a higher colistin MIC (>16 μg/mL) than that previously observed in all tests, thus demonstrating the presence of an *A. baumannii* subpopulation that turned out to be resistant to colistin.

## 3. Discussion

The persistence in hospital environments and the extraordinary ability to rapidly acquire resistance to new antibiotics rendered problematic the treatment and eradication of infections caused by multidrug-resistant (MDR) *A. baumannii* strains [20]. In this dramatic situation, colistin often represents the last-resort (lifesaving) drug for the treatment of infections sustained by *A. baumannii* [21,22]. However, since 1999 there has been evidence of the growing frequency of nosocomial *A. baumannii* isolates that showed resistance to colistin [23]. In this work, the vast majority of *A. baumannii* isolates obtained from ICU patients were classified as XDR (86.27%) or PDR (11.76%) [18], witnessing the evolution of *A. baumannii* towards panresistance and causing serious alarm about the increasing prevalence of colistin-resistant *A. baumannii*, especially in ICU settings [18,19].

A progressive decline in susceptibility to β-lactam antibiotics in *A. baumannii* has been documented over the past several decades [24]. Carbapenem resistance was mainly attributed to the spread of genes encoding for oxacillinase (*bla*_OXA-like_). In the early 2000s, OXA-58 oxacillinases were the most widespread in Italy and neighboring countries, but their prevalence has drastically decreased over the years, with the onset of OXA-23, which gradually replaced OXA-58 [25]. Therefore, the epidemiological profiles of the carbapenem-resistant *A. baumannii* isolates analyzed in the present work are largely consistent with the traits of MDR *A. baumannii* circulating in Central Italy since 2005 [7,26,27,28], as documented by the high prevalence (86.28%) of isolates belonging or related to ICL II, carrying the *bla*_OXA-23_ carbapenemase gene. By comparison with previous epidemiological data, major differences are observed in the emergence of a new clone in Central Italy, consisting in ICL I strains carrying the *bla_OXA-24/40_* gene (13.72% of the isolates). It seems therefore that ICL I re-emerged in Central Italy after a decade predominated by ICL II [26] in association with the *bla*_OXA-24/40_ gene that has anecdotally been documented in Italy, invariably associated with ICL II [29]. These findings provide useful updates about the prevalence of XDR and PDR *A. baumannii* in Central Italy, and highlight the emergence of novel, potentially epidemic, clones.

The problem of the clinical management of patients affected by PDR *A. baumannii* strains poses a number of questions about the correct execution and interpretation of colistin susceptibility tests, especially agar diffusion tests (e.g., E-test^®^, disk-diffusion) and automated systems (e.g., Vitek^®^2). The relative instability of the maintenance of concentration gradients of colistin in agar plates has raised a caveat against the use of such systems, both manual and automated. In fact, isolates that were considered susceptible to the action of the antibiotic resulted resistant when tested with reference tests, such as broth dilution methods [11]. Inappropriate colistin susceptibility testing can lead to misinterpretation of the results and, consequently, to an inadequate antibiotic therapy [11]. For this reason, the susceptibility to colistin of all of the isolates of our collection was compared between broadly used commercial methods, namely Vitek^®^ 2 and MIC Test Strips, and the broth microdilution method, considered as the gold standard. Data revealed from comparison of the three methods indicate poor concordance between Vitek^®^2 or MIC Test Strips and the broth microdilution method (<90% essential agreement). The MIC values obtained through both commercial methods were discordant by ca. 10% when compared with the MIC values obtained by the broth microdilution, even setting the MIC discordancy threshold at more than two dilution factors. These results corroborate the conclusion that diffusion methods should not be used to test colistin susceptibility in *A. baumannii* [11] and make questionable colistin susceptibility testing using Vitek^®^ 2 [30]. Intriguingly, all of the 13 strains that showed discordant values between Vitek^®^2 and the reference broth microdilution method were found to belong to ICL II. Of course, this observation is far from a general conclusion and further studies are needed to confirm this result.

In time-course experiments, we observed that the growth in the presence of colistin of a subset of *A. baumannii* isolates whose MIC at Vitek^®^2 showed values close to the sensitivity breakpoint (MIC ≤ 2 μg/mL) became spectrophotometrically detectable only after at least 12 h of incubation. To the best of our knowledge, no other sound studies attempted to correlate the kinetic of growth of *A. baumannii* in the presence of colistin in a microdilution method at concentrations that are critical in discriminating resistance or susceptibility of a given strain. In our opinion, if discontinuity of the concentration gradient of colistin diffusion in the agar explains the inaccuracy of the diffusion tests, a different explanation should be applied to the results obtained with the Vitek^®^ 2 system. In fact, this is an instrument with a temperature-controlled incubation chamber (35.5 °C), which, to provide early information on the sensitivity of an antibiotic, signals bacterial growth as soon as it is detected (at approximately four hours). Therefore, the duration of the incubation time could represent an important limiting factor in determining the accuracy of colistin MIC, since microbial growth, which starts after about 10–12 h at >2 μg/mL (breakpoint) antibiotic concentrations, could be undetectable at shorter incubation times. This could explain why in our comparative tests the Vitek^®^2 system underestimated MIC levels for colistin, resulting in problematic therapeutic implications.

To make the picture more complicated, we observed the presence of heteroresistance to colistin in several isolates of *A. baumannii.* Although the population analysis profile (PAP) seems to show a better accuracy in detecting heteroresistance by quantifying the proportion of resistant cells existing within a culture, in this work we adopted the E-test^®^ approach that has been shown to correctly identify heteroresistant subpopulations in a faster and less time-consuming manner [31]. Our results show the presence of heteroresistance in three out of the six strains that presented discordance in the susceptibility to colistin. Therefore, it is our opinion that heteroresistance cannot be considered a confunding factor in determining the extent of susceptibility to colistin in our strain collection.

In conclusion, this study highlights that the high frequency of isolation of XDR and PDR *A. baumannii* isolates in high-risk infectious wards requires an accurate application of methods for detecting susceptibility to antibiotics, in particular to last-resort antibiotics like colistin, so to ensure a correct therapeutic approach.

## 4. Materials and Methods

Strains of *A. baumannii* (*n* = 51) were isolated from clinical samples of 32 patients admitted from February 2017 until July 2017 to the ICU of a 1300-bed tertiary care academic hospital (Policlinico “Umberto I”, Rome, Italy) with an average acceptance of 38,000 patients/year. The clinical samples considered in this study were respiratory secretions, blood cultures, urine, central venous catheters, and samples from other sites (i.e.,cerebrospinal fluid (CSF), nasal and wound swabs, wound drainage, abscess). The samples were cultured on agar media (blood agar and MacConkey Agar, bioMérieux, Marcy l’Etoile, France) and incubated for 24 h at 37 °C. The bacterial colonies were identified by a semiautomatic biochemical method (ID-GN cards, Vitek^®^2, bioMérieux) and MALDI-TOF mass spectrophotometry (Bruker Daltonics, Bremen, Germany), with a discriminatory score >2300 [32].

The MIC of various antibiotics tested (amoxicillin/clavulanic acid, piperacillin/tazobactam, cefotaxime, ertapenem, imipenem, meropenem, gentamicin, ciprofloxacin, tigecycline, fosfomycin, nitrofurantoin, colistin, and trimethoprim/sulfamethoxazole) was assayed by different methods on isolated colonies of *A. baumannii*, resuspended in 0.45% NaCl solution at an inoculum concentration of 0.50 McFarland (MF). In the first instance, antimicrobial susceptibility profiles were tested using the Vitek^®^2 system (AST N-202 card, bioMérieux).

Multiplex PCR was performed to identify resistance genes belonging to oxacillinases (*bla*_OXA-like_). The sequences of *bla*_OXA-like_ alleles encoding carbapenemases were aligned and group-specific regions were identified using Bioedit software [33]. Primer pair 5-TAA TGC TTT GAT CGG CCT TG and 5-TGG ATT GCA CTT CAT CTT GG was used to amplify a 353 bp fragment of genes encoding the intrinsic OXA-51-like enzymes of *A. baumannii*. These primers were combined with six new primers that were designed to amplify fragments of genes encoding acquired OXA-23-like (501 bp: 5-GAT CGG ATT GGA GAA CCA GA and 5-ATT TCT GAC CGC ATT TCC AT), OXA-24-like (246 bp: 5-GGT TAG TTG GCC CCC TTA AA and 5-AGT TGA GCG AAA AGG GGA TT) and OXA-58-like (599 bp: 5-AAG TAT TGG GGC TTG TGC TG and 5-CCC CTC TGC GCT CTA CAT AC), OXA-143-like (149 bp: 5-TGG CAC TTT CAG CAG TTC CT and 5-TAA TCT TGA GGG GGC CAA CC) carbapenemases (Table 2). The amplification conditions were initial denaturation at 94 °C for 5 min, 30 cycles of 94 °C for 25 s, 52 °C for 40 s, and 72 °C for 50 s, and a final elongation at 72 °C for 6 min [34,35].

Several molecular methods with different resolutions have been used to type *A. baumannii* strains [9]. A typing scheme based on two multiplex PCRs targeting three genes under selective pressure (*ompA*, *csuE*, and *bla*_OXA-51-like_) has been a convenient method for rapid assignment of *A. baumannii* isolates into three major PCR-based groups (Gs) corresponding to international clones I (G2), II (G1), and III (G3) [19]. Multiplex PCRs for identification of the *ompA*, *csuE*, and *bla*_OXA-51-like_ sequence groups defined as Group 1 and Group 2 were performed using the primers listed in Table 3. Primers for *ompA* and *csuE* were designed from sequences available at GenBank (AY485227, DQ093960; AY241696) or from sequences determined during initial studies (DQ289014–DQ289019). These were aligned and consensus primers were designed from common sequence areas. The amplification conditions were: 94 °C for 3 min, followed by 30 cycles of 94 °C for 45 s, 57 °C for 45 s, and 72 °C for 1 min, followed by a final extension at 72 °C for 5 min [19,36]. Identification of a strain as a member of Group 1 or Group 2 required the amplification of all three fragments in the corresponding multiplex PCR, and an absence of any amplification by the other multiplex PCR. Group 3 isolates were defined by the amplification of only the *ompA* fragment in the Group 2 PCR, and the amplification of only the *csuE* and *bla*_OXA-51-like_ fragments in the Group 1 PCR.

Subsequently, for further confirmations of the MICs for colistin, the methods for concentration gradient diffusion (MIC Test Strips, Liofilchem srl, Italy) and the broth microdilution method (Customized stripes, Sensititre, Thermo Diagnostic Systems, East Grinstead, Great Britain) were used. Broth microdilution was carried out inoculating 100 μL of a bacterial suspension at a final concentration of 5 × 10^5^ CFU/mL in the wells of a sterile round bottom polystyrene microplate, according to the manufacturer’s instructions. Colistin panel concentrations ranged between 0.12 and 8 μg/mL. MIC determination for colistin by MIC Test Strips, with concentrations ranging from 0.016 to 256 μg/mL, was performed on Mueller–Hinton Agar II plates (bioMérieux, Marcy l’Etoile, France), smoothing out the starting inoculum (0.50 MF). In all cases, the microbial cultures were incubated at 37 °C for 18–24 h.

The latest EUCAST interpretative criteria of the MICs for colistin were used [9], considering resistant the strains with an MIC ≥ 4 μg/mL. In all the experiments, *Escherichia coli* ATCC 25922 was used as the susceptible control strain. Furthermore, *A. baumannii* type strain ATCC 19606 was used as colistin-susceptible control strain.

The growth dynamics of 13 *A. baumannii* strains in the presence of colistin scalar concentrations were kinetically tested in sterile microplates using a microtiter plate reader (Spark, Tecan Group, Mannedorf, Switzerland). After checking the 0.50 MF inoculum by the reference standard, the sample absorbance was automatically detected at OD_600_ at two-hour intervals for 24 h and at a temperature of 37 °C. The results were used to construct the bacterial growth curves, correlating the absorbance in function of time.

The presence of colistin heteroresistance in six isolates of *A. baumannii* was checked by plating the bacterial suspension at 0.50 MF and 2 MF on Mueller–Hinton Agar II plates and tested with MIC Test Strips at 37 °C for 48 h.

## Figures and Tables

**Table 1:** Source of the isolates in the patient population.

N. Patient	Id. Isolate	Source	N. Patient	Id. Isolate	Source
**1**	#1	Respiratory Secretion	**16**	#26	Respiratory Secretion
	#2	Blood Colture	**17**	#27	Wound Swab
**2**	#3	Blood Colture	**18**	#28	Respiratory Secretion
**3**	#4	Respiratory Secretion	**19**	#29	Respiratory Secretion
**4**	#5	Respiratory Secretion	**20**	#30	Sputum
**5**	#6	Blood Colture	**21**	#31	Nasal Swab
	#7	Respiratory Secretion		#32	Blood Colture
**6**	#8	Respiratory Secretion		#33	Respiratory Secretion
	#9	Blood Colture	**22**	#34	Respiratory Secretion
**7**	#10	Blood Colture		#35	Blood Colture
	#11	Respiratory Secretion	**23**	#36	Respiratory Secretion
	#12	Central Venous Catheter	**24**	#37	Blood Colture
**8**	#13	Blood Colture	**25**	#38	Respiratory Secretion
**9**	#14	Respiratory Secretion	**26**	#39	Respiratory Secretion
**10**	#15	Blood Colture	**27**	#40	Blood Colture
	#16	Wound Swab	**28**	#41	Respiratory Secretion
**11**	#17	Respiratory Secretion	**29**	#42	Blood Colture
**12**	#18	Respiratory Secretion	**30**	#43	Cerebrospinal fluid
**13**	#19	Urine		#44	Blood Colture
	#20	Respiratory Secretion		#45	Central Venous Catheter
	#21	Blood Colture		#46	Respiratory Secretion
**14**	#22	Blood Colture	**31**	#47	Blood Colture
	#23	Respiratory Secretion	**32**	#48	Ascessive Liquid
**15**	#24	Respiratory Secretion		#49	Urine
	#25	Central Venous Catheter		#50	Blood Colture
				#51	Respiratory Secretion

**Table 2:** Primers used for PCR amplification of oxacillinase resistance genes (bla_OXA-like_).

Primer	Sequence (5′–3′)	Target	Amplicon Size (bp)
*bla*_OXA-51_ FW	TAA TGC TTT GAT CGG CCT TG	*bla*_OXA-51-like_	353
*bla*_OXA-51_ RV	TGG ATT GCA CTT CAT CTT GG		
*bla*_OXA-23_ FW	GAT CGG ATT GGA GAA CCA GA	*bla*_OXA-23-like_	501
*bla*_OXA-23_ RV	ATT TCT GAC CGC ATT TCC AT		
*bla*_OXA-24_ FW	GGT TAG TTG GCC CCC TTA AA	*bla*_OXA-24-like_	246
*bla*_OXA-24_ RV	AGT TGA GCG AAA AGG GGA TT		
*bla*_OXA-58_ FW	AAG TAT TGG GGC TTG TGC TG	*bla*_OXA-58-like_	599
*bla*_OXA-58_ RV	CCC CTC TGC GCT CTA CAT AC		
*bla*_OXA-143_ FW	TGG CAC TTT CAG CAG TTC CT	*bla*_OXA-143-like_	149
*bla*_OXA-143_ RV	TAA TCT TGA GGG GGC CAA CC		

**Table 3:** Primers used in multiplex PCRs for identification of international clonal lineages.

Primer	Sequence (5’–3’)	Target	Amplicon Size (bp)
Group1ompAF306	GAT GGC GTA AAT CGT GGT A	*ompA*	355
Group1and2ompAR660	CAA CTT TAG CGA TTT CTG G		
Group1csuEF	CTT TAG CAA ACA TGA CCT ACC	*csuE*	702
Group1csuER	TAC ACC CGG GTT AAT CGT		
Gp1OXA66F89	GCG CTT CAA AAT CTG ATG TA	*bla*_OXA-51-like_	559
Gp1OXA66R647	GCG TAT ATT TTG TTT CCA TTC		
Group2ompAF378	GAC CTT TCT TAT CAC AAC GA	*ompA*	343
Group1and2ompAR660	CAA CTT TAG CGA TTT CTG G		
Group2csuEF	GGC GAA CAT GAC CTA TTT	*csuE*	580
Group2csuER	CTT CAT GGC TCG TTG GTT		
Gp2OXA69F169	CAT CAA GGT CAA ACT CAA	*bla*_OXA-51-like_	162
Gp2OXA69R330	TAG CCT TTT TTC CCC ATC		

## Data Availability

Data sharing is not applicable to this article.

## Supplementary Materials

The following are available online at https://www.mdpi.com/2079-6382/10/1/48/s1, Table S1: Distribution isolates by sample; Table S2: Distribution of isolates according to infections and colonization; Table S3: Antimicrobial susceptibility profile of *A. baumannii* strains according to Vitek^®^2; Table S4: MICs for colistin determined by MIC Test Strip, Vitek^®^2, and broth microdilution.
