# Supplementary material for: Susceptibility Testing of Colistin for Acinetobacter baumannii: How Far Are We from the Truth?
Source: Antibiotics (Basel). 2021 Jan 5;10(1):48. doi: 10.3390/antibiotics10010048 (PMC7824894; doi:10.3390/antibiotics10010048)
Supplement: Supplementary file 1 [file antibiotics-10-00048-s001.pdf]

antibiotics

MDPI

Article

Susceptibility Testing of Colistin for *Acinetobacter baumannii*: How Far are We from the Truth?

Federica Sacco [1,2], Paolo Visca [3], Federica Runci [3], Guido Antonelli [2,4] and Giammarco Raponi [2,5,*]

[1] Department of Molecular Medicine, University of Rome "La Sapienza", 00161 Rome, Italy; federica.sacco@uniroma1.it (F.S.)
[2] Clinical Microbiology Laboratory, University Hospital Policlinico Umberto I of Rome, 00185 Rome, Italy; federica.sacco@uniroma1.it (F.S.); guido.antonelli@uniroma1.it (G.A.)
[3] Department of Science, University of Rome "Roma Tre", 00154 Rome, Italy; paolo.visca@uniroma3.it (P.V.); federica.runci@uniroma3.it (F.R.)
[4] Department of Molecular Medicine, Laboratory of Virology and Pasteur Institute-Cenci Bolognetti Foundation, 00161 Rome, Italy
[5] Department of Public Health Sciences and Infectious Diseases, 00185 Rome, Italy
* Correspondence: giammarco.raponi@uniroma1.it; Tel.: +39-06-4991-4616.

Table S1. Distribution isolates by sample.

Isolation Sites	Strains (%)
Respiratory samples	24 (47.06%)
Blood cultures	17 (33.33%)
Central venous catheters	3 (5.88%)
Urine	2 (3.92%)
Wound swab	2 (3.93%)
Abscess liquid	1 (1.96%)
CSF	1 (1.96%)
Nasal swab	1 (1.96%)

Table S2. Distribution of isolates according to infections and colonization.

Origin of the Strains	Strains (%)
Bacteraemia	13 (25.48%)
Bacteraemia CVC-correlated	2 (3.92%)
Ventilator-associated pneumonia	19 (37.26%)
Infections from other sites	5 (9.81%)
Respiratory colonization	5 (9.81%)
Colonization of the other sites	7 (13.72%)

Table S3. Antimicrobial susceptibility profile of *A. baumannii* isolates according to Vitek®2.

Isolate Id.	Antimicrobial*											
	A/AA	P/T	CTX	EPM	IMP	MEM	GM	CIP	TGC	FOS	CST	T/S
#1	R	R	R	R	R	R	R	R	R	R	S	R
#2	R	R	R	R	R	R	R	R	R	R	S	R
#3	R	R	R	R	R	R	R	R	R	R	S	R
#4	R	R	R	R	R	R	R	R	R	R	S	R
#5	R	R	R	R	R	R	R	R	R	R	S	R
#6	R	R	R	R	R	R	R	R	R	R	S	R
#7	R	R	R	R	R	R	R	R	R	R	S	R
#8	R	R	R	R	R	R	R	R	R	R	S	R
#9	R	R	R	R	R	R	R	R	R	R	S	R
#10	R	R	R	R	R	R	R	R	R	R	S	R
#11	R	R	R	R	R	R	R	R	R	R	S	R
#12	R	R	R	R	R	R	R	R	R	R	S	R
#13	R	R	R	R	R	R	R	R	R	R	S	R
#14	R	R	R	R	R	R	R	R	S	R	S	R
#15	R	R	R	R	R	R	R	R	S	R	S	R
#16	R	R	R	R	R	R	R	R	R	R	S	R
#17	R	R	R	R	R	R	R	R	R	R	S	R
#18	R	R	R	R	R	R	R	R	R	R	S	R
#19	R	R	R	R	R	R	R	R	R	R	S	R
#20	R	R	R	R	R	R	R	R	R	R	S	R
#21	R	R	R	R	R	R	R	R	R	R	S	R
#22	R	R	R	R	R	R	R	R	R	R	S	R
#23	R	R	R	R	R	R	R	R	R	R	S	R
#24	R	R	R	R	R	R	R	R	R	R	R	R
#25	R	R	R	R	R	R	R	R	R	R	S	R
#26	R	R	R	R	R	R	R	R	R	R	S	R
#27	R	R	R	R	R	R	R	R	R	R	R	R
#28	R	R	R	R	R	R	R	R	R	R	S	R
#29	R	R	R	R	R	R	R	R	R	R	S	R
#30	R	R	R	R	R	R	R	R	R	R	S	R
#31	R	R	R	R	R	R	R	R	S	R	S	R
#32	R	R	R	R	R	R	R	R	R	R	S	R
#33	R	R	R	R	R	R	R	R	R	R	S	R
#34	R	R	R	R	R	R	R	R	R	R	R	R
#35	R	R	R	R	R	R	R	R	R	R	R	R
#36	R	R	R	R	R	R	R	R	S	R	S	R
#37	R	R	R	R	R	R	R	R	S	R	S	R
#38	R	R	R	R	R	R	R	R	R	R	S	R
#39	R	R	R	R	R	R	R	R	R	R	S	R
#40	R	R	R	R	R	R	R	R	R	R	S	R
#41	R	R	R	R	R	R	R	R	R	R	S	R
#42	R	R	R	R	R	R	R	R	R	R	S	R
#43	R	R	R	R	R	R	R	R	R	R	S	R
#44	R	R	R	R	R	R	R	R	R	R	S	R
#45	R	R	R	R	R	R	R	R	R	R	S	R
#46	R	R	R	R	R	R	R	R	R	R	S	R
#47	R	R	R	R	R	R	R	R	S	R	S	R
#48	R	R	R	R	R	R	R	R	R	R	R	R
#49	R	R	R	R	R	R	R	R	R	R	S	R
#50	R	R	R	R	R	R	R	R	S	R	S	R
#51	R	R	R	R	R	R	R	R	R	R	R	R
**Susceptibility (%)**	**0**	**0**	**0**	**0**	**0**	**0**	**0**	**0**	**13.7**	**0**	**88.2**	**0**

*Abbreviations are according to the Vitek®2 nomenclature: A/AA, amoxicillin/clavulanic acid; P/T, piperacillin/tazobactam; CTX, cefotaxime; EPM, ertapenem; IMP, imipenem; MEM, meropenem; GM, gentamicin; CIP, ciprofloxacin; TGC, tigecycline; FOS, fosfomycin; CST, colistin; T/S, trimethoprim/sulfamethoxazole. Grey-filled boxes denote susceptibility.

Table S4. MICs for colistin determined by MIC Test Strip, Vitek®2 and broth microdilution.

Isolate Id.	MIC [µg/mL]*		
	Test Strip	VITEK®2	Broth Microdilution
#1	1.50	2	1
#2	3	2	1
#3	4	**2**	**8**
#4	2	2	1
#5	2	≤0.50	0.50
#6	**6**	**2**	4
#7	**8**	**2**	**8**
#8	0.75	≤0.50	< 0.12
#9	**4**	**≤0.50**	0.50
#10	1.5	≤0.50	0.50
#11	0.75	≤0.50	0.50
#12	0.50	≤0.50	<0.50
#13	1	≤0.50	<0.12
#14	0.50	≤0.50	0.50
#15	1	≤0.50	1
#16	1.50	≤0.50	1
#17	0.75	≤0.50	0.50
#18	0.75	≤0.50	0.50
#19	1	≤0.50	0.50
#20	0.75	≤0.50	0.50
#21	0.75	≤0.50	0.25
#22	0.50	≤0.50	0.50
#23	2	≤0.50	0.50
#24	4	16	>8
#25	**16**	**≤0.50**	**>8**
#26	0.50	≤0.50	0.50
#27	**1**	**8**	4
#28	**12**	**2**	**8**
#29	3	2	1
#30	1.50	≤0.50	0.50
#31	1.50	≤0.50	1
#32	1.50	≤0.50	1
#33	1.50	≤0.50	1
#34	8	4	4
#35	16	8	8
#36	0.75	≤0.50	0.50
#37	1.50	≤0.50	0.50
#38	3	≤0.50	0.50
#39	0.75	≤0.50	0.50
#40	0.75	≤0.50	0.50
#41	2	≤0.50	1
#42	1	1	1
#43	0.75	≤0.50	0.50
#44	2	≤0.50	0.50
#45	2	≤0.50	1
#46	**4**	**≤0.50**	**8**
#47	0.75	≤0.50	1
#48	6	**4**	**>8**
#49	3	≤0.50	0.50
#50	3	≤0.50	0.50
#51	16	8	>8

*Resistance values are grey shaded; discordant MIC values are in bold.
